# Sexual dysfunction in women with breast cancer: a systematic review

**DOI:** 10.1007/s00520-025-09352-6

**Published:** 2025-03-31

**Authors:** Nelson Rodrigues-Machado, Xavier Bonfill-Cosp, M. Jesús Quintana, Marilina Santero, Ana Bártolo, Anna Selva Olid

**Affiliations:** 1 Methodology of Biomedical Research and Public Health, Universitat Autònoma de Barcelona, Bellaterra, Spain; 2 Research Group on Chronic Care and Health Innovation (GRACIS), Department of Health Sciences, Tecnocampus, Pompeu Fabra University, Mataró, Spain; 3 Universitat Autònoma de Barcelona, Barcelona, Spain; 4 Institut de Recerca Sant Pau (IR Sant Pau), Barcelona, Spain; 5 Centro de Investigación Biomédica en Red de Epidemiología y Salud Pública (CIBERESP), Madrid, Spain; 6 Centro Cochrane Iberoamérica, Barcelona, Spain; 7 CINTESIS@RISE, CINTESIS.UPT, Portucalense University, Porto, Portugal; 8 Clinical Epidemiology and Cancer Screening, Parc Taulí Hospital Universitari, Institut d’Investigació I Innovació Parc Taulí (I3PT_CERCA), Universitat Autònoma de Barcelona, Sabadell, Spain

**Keywords:** Breast cancer, Sexual dysfunction, Prevalence, Risk factors, Systematic review, Survivorship

## Abstract

**Purpose:**

This systematic review aims to assess the prevalence, incidence, and risk factors for sexual dysfunction (SdF) in women with breast cancer (BC).

**Methods:**

A systematic search was conducted in MEDLINE (PubMed), PsycINFO, Web of Science, and CINAHL to identity longitudinal, observational studies assessing SdF in women with BC. Data extraction followed PRISMA guidelines. The Joanna Briggs Institute checklist was used to assess methodological quality. Results were narratively synthesised.

**Results:**

A total of 16 studies involving 4058 women met the inclusion criteria. Overall, the reported prevalence of SdF ranged from 17.5% before BC diagnosis to 86% after 6 months of hormone therapy. Only one study reported incidence data. The six most commonly studied SdF domains were desire, arousal, lubrication, orgasm, satisfaction, and dyspareunia. A significant number of risk factors associated with different dimensions of SdF were identified.

**Conclusion:**

SdF is highly prevalent in women with BC, particularly in the first year after diagnosis and treatment. These findings strongly suggest that SdF should be routinely assessed as part of survivorship care protocols. Due to the scant data on incidence rates and the wide variability in reported risk factors, significant gaps remain in our understanding of the onset and progression of SdF in patients with BC. Well-designed cohort studies are needed to better establish the incidence and aetiology of SdF in this patient population.

**Supplementary Information:**

The online version contains supplementary material available at 10.1007/s00520-025-09352-6.

## Introduction

Breast cancer (BC) is the most common type of cancer in women and the leading cause of cancer-related mortality in females [1]. Although treatment outcomes can be highly variable depending on the country [1, 2], survival rates have markedly improved in recent years, mainly due to the widespread implementation of screening mammography and therapeutic advancements. This improvement in survival rates has led to a growing interest in identifying the factors that impact quality of life (QoL) in these patients [3, 4].

One factor that can have a large negative impact on QoL in BC survivors is sexual dysfunction (SdF), a complex, multidimensional issue that can be challenging to manage [5]. SdF encompasses a range of difficulties experienced during the sexual response cycle that hinder satisfaction from sexual activity and women often experience multiple types of SdF concurrently [6, 7]. SdF is typically assessed through the use of specific instruments designed to assess the different domains of sexual function.

Compared to the general population, women with BC face an elevated risk of developing SdF at all stages of survivorship, the presence of which can pose significant challenges to sexual QoL [8–11]. SdF comprises a wide range of issues, including penetration pain, decreased libido, lubrication problems, dysfunctional excitement, vaginal dryness, and dyspareunia, with the two latter symptoms being particularly common. Other factors that may influence the risk and severity of SdF include the length of the marriage, pretreatment sexual health concerns, endocrine therapy, duration of chemotherapy (ChT), emotional distress, comorbidities, and type of surgery [12–15].

Despite the growing interest in obtaining a better comprehension of SdF in BC survivors, most systematic reviews performed to date have predominantly focused on cross-sectional studies, thereby limiting our understanding of the temporal evolution of SdF [16–18]. Moreover, some reviews have used general QoL instruments to assess SdF, which may not capture the full scope and complexity of this condition [19]. As a result, there are significant knowledge gaps in our understanding of SdF in this specific patient population, thus underscoring the need for a more comprehensive longitudinal approach. Therefore, we conducted this systematic review based on longitudinal studies in order to determine the incidence and prevalence of SdF in women diagnosed with BC, to describe changes in SdF over time, and to identify potential risk factors in this patient population.

## Methods

This systematic review adhered to the *Cochrane Handbook of Systemic Reviews of Interventions* [20] and results are reported in accordance with the Preferred Reporting Items for Systematic reviews and Meta-Analysis (PRISMA) 2020 statement [21]. The study protocol is publicly available on the Open Science Framework (https://osf.io/ysf4b/).

### Eligibility criteria

We included peer-reviewed, longitudinal observational studies that used validated instruments to assess SdF in adult (≥ 18 years) women diagnosed with BC. The minimum sample size was 50. Studies published between January 2010 and November 2023 in English, Spanish, or Portuguese were eligible for inclusion. Randomized clinical trials, case-control, and cross-sectional studies, reviews, conference and poster abstracts, pilot studies, commentaries, dissertations, editorials, and summary reports were excluded.

### Information sources

We systematically searched MEDLINE (via PubMed), PsycINFO, Web of Science, and *the Cumulative Index to Nursing & Allied Health Literature* (CINAHL) through November 30, 2023. The comprehensive search strategy combined MeSH terms and free-text words related to “breast cancer” and “sexual dysfunction”, including specific SdF domains. Details of the MEDLINE search are shown in Online Resource S1.

### Data extraction and synthesis

The search results were downloaded into the Covidence® software program. First, we removed all duplicates and then three independent researchers (NR, AB, MS) screened the titles and abstracts in pairs to identify potentially eligible studies. The same researchers then evaluated the full texts of studies that appeared to meet the inclusion criteria. Disagreements were resolved by consensus among the three researchers. We developed and pilot tested a data extraction form to collect key data for each article, including author, year of publication, country, study design, sample size, age (mean/median and standard deviation [SD] or interquartile range), questionnaire(s) used, incidence and prevalence of SdF, assessment time points, sexual function outcomes (overall and by domain), potential risk factors, and measures of association (with 95% confidence intervals [CI] and *p*-values). SdF was defined as either the total score on the instrument or the presence of at least one SdF, even if the domain was not specified.

Given the heterogeneous results of these studies, it was not feasible to perform a meta-analysis. For this reason, we performed a narrative synthesis instead. Tables and figures were created to synthesize the results and to facilitate data interpretation. We plotted the prevalence data and the mean/median SdF scores (overall and by domain) at different time points for each study.

### Quality assessment

Two authors independently assessed the methodological quality of the studies using the Joanna Briggs Institute critical appraisal checklist for cohort studies [22]. Disagreements were resolved by consensus. This appraisal tool evaluates 11 key quality criteria, with possible responses of “yes”, “no”, “unclear”, or “not applicable”. The overall score for each study was determined by the number of “yes” responses, with higher scores indicating lower risk of bias.

## Results

**Fig. 1:**
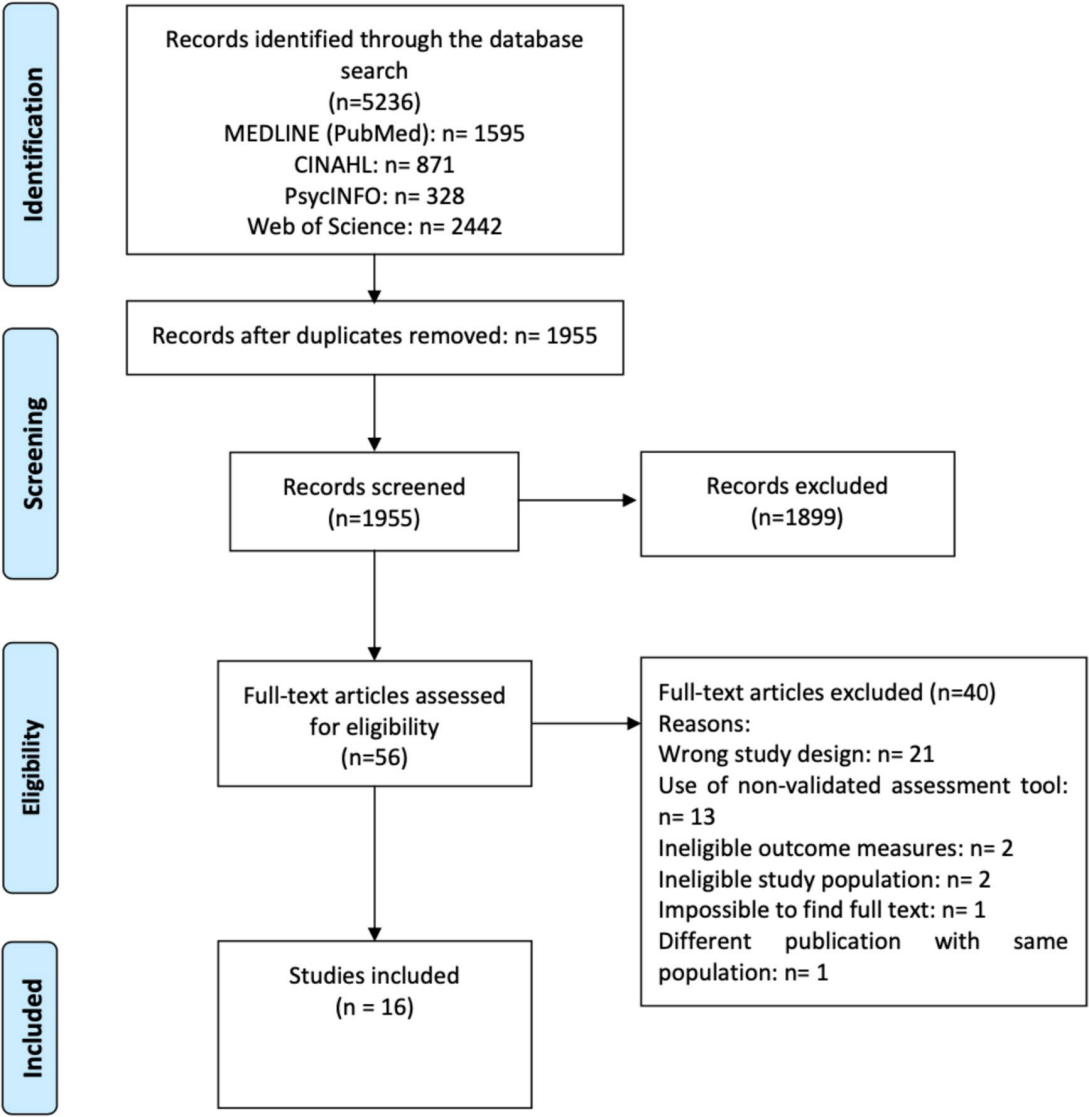
PRISMA flowchart

The search yielded 5236 references. Of these, 16 studies met the inclusion criteria (Fig. 1).

Most of the studies (*n* = 10) used a one-group cohort design, with the remaining studies (*n* = 6) using two groups. The 16 studies comprised a total of 4058 women with BC from 10 different countries (USA, Spain, Canada, Germany, Iran, Turkey, South Korea, Denmark, Sweden, and Australia). These studies used 11 different validated instruments to assess SdF, most commonly the Female Sexual Function Index (FSFI) [24–28, 30] and the Sexual Activity Questionnaire (SAQ) [26, 31, 34] (Table 1).

**Table 1:** Characteristics of the 16 studies included in the systematic review

Author, year	Country	Study design	*N*/CG	Age, years: mean (± SD) or median (range)	Quality assessment instrument	Risk of bias
Bober et al., 2013 [23]	USA	Single cohort study	304	50.3 (26–84)	WSFQ	63.6%
Córdoba-de Juan et al., 2019 [24]	Spain	Single cohort study	89	56.89 (± 11.45)	FSFI	45.5%
Cornell et al., 2017 [25]	USA	Cohort study	226	56 (28–79)	FSFI	72.2%
Farthmann et al., 2016 [26]	Germany	Cohort study	79	47.46 (± 9.75), range: 23–70	SAQ FSFI	72.2%
Frechette et al., 2013 [27]	Canada	Single Cohort study	66	61 (50–80)	FSFI FSDS-R	63.6%
Harirchi et al., 2012 [28]	Iran	Single cohort study	216	44.3 (± 8.6)	FSFI	63.6%
Ïzci et al., 2020 [29]	Turkey	Cohort study	56/52	53 (± 33) CG: 52.5 (± 33)	ASEX	81.8%
Lee et al., 2015 [30]	South Korea	Single cohort study	304	46.0 (23–57)	FSFI	63.6%
Metcalfe et al., 2012 [31]	Canada	Cohort study	190	Mastectomy alone: 53.5 (30–83) Mastectomy with immediate reconstruction: 46.2 (28–66) Delayed reconstruction: 51.6 (33–78)	SAQ	72.2%
Rosenberg et al., 2020 [32]	USA	Cohort study	826	BCS: 35.9 UM: 36.4 BM: 36.1	CARES	72.2%
Rottmann et al., 2017 [33]	Denmark	Single cohort study	287	55.77 (± 10.01)	PROMIS SexFS, v. 1.0	63.6%
Unukovych et al., 2012 [34]	Sweden	Single cohort study	60	NR	SAQ	54.5%
Vaidakis et al., 2014 [35]	Greece	Cohort study	67/33	50.5 (± 6.8)	SFQ	81.8%
Verma et al., 2022 [36]	USA	Single cohort study	300	62.4 (± 11.0)	MOS-Sexual Problems	54.5%
Von Hippel et al., 2019 [37]	USA	Single cohort study	896	35.9 (± 4.0), range: 18–40	CARES	54.5%
Webber et al., 2011 [38]	Australia	Single cohort study	92	49.8 (± 8.8)	CARES	54.5%

Abbreviations: *BCS* breast-conserving surgery, *BMR* bilateral mastectomy with reconstruction, *BMWR* bilateral mastectomy without reconstruction, *CG* control group, *NR* not reported, *UMR* unilateral mastectomy with reconstruction, *UMWR* unilateral mastectomy without reconstruction, *USA* United States of America, *FSFI* Female Sexual Function Index, *SAQ* Sexual Activity Questionnaire, *WSFQ* Watts Sexual Function Questionnaire, *FSDS-R* Female Sexual Distress Scale-Revised, *CARES* Cancer Rehabilitation Evaluation System, *ASEX* Arizona Sexual Life Scale, *SFQ* Sexual Function Questionnaire, *MOS* Medical Outcomes Study, *PROMIS-SexFS* Patient-Reported Outcomes Measurement Information System Sexual function and satisfaction

### Methodological quality assessment

The final quality assessment scores are shown in Table 1. Additional details are available in Online Resource S2. Overall, the methodological quality of the studies was highly variable. Although most of the studies used validated, reliable outcome measures, with sufficient follow-up and appropriate statistical analysis, we identified several methodological weaknesses. For example, none of the studies provided clear evidence that participants did not present SdF at the start of the study. In addition, many studies did not clearly identify confounding factors. Finally, the strategies used to address incomplete follow-up data were often unclear or not described.

### Sexual dysfunction

#### Overall sexual dysfunction

**Fig. 2:**
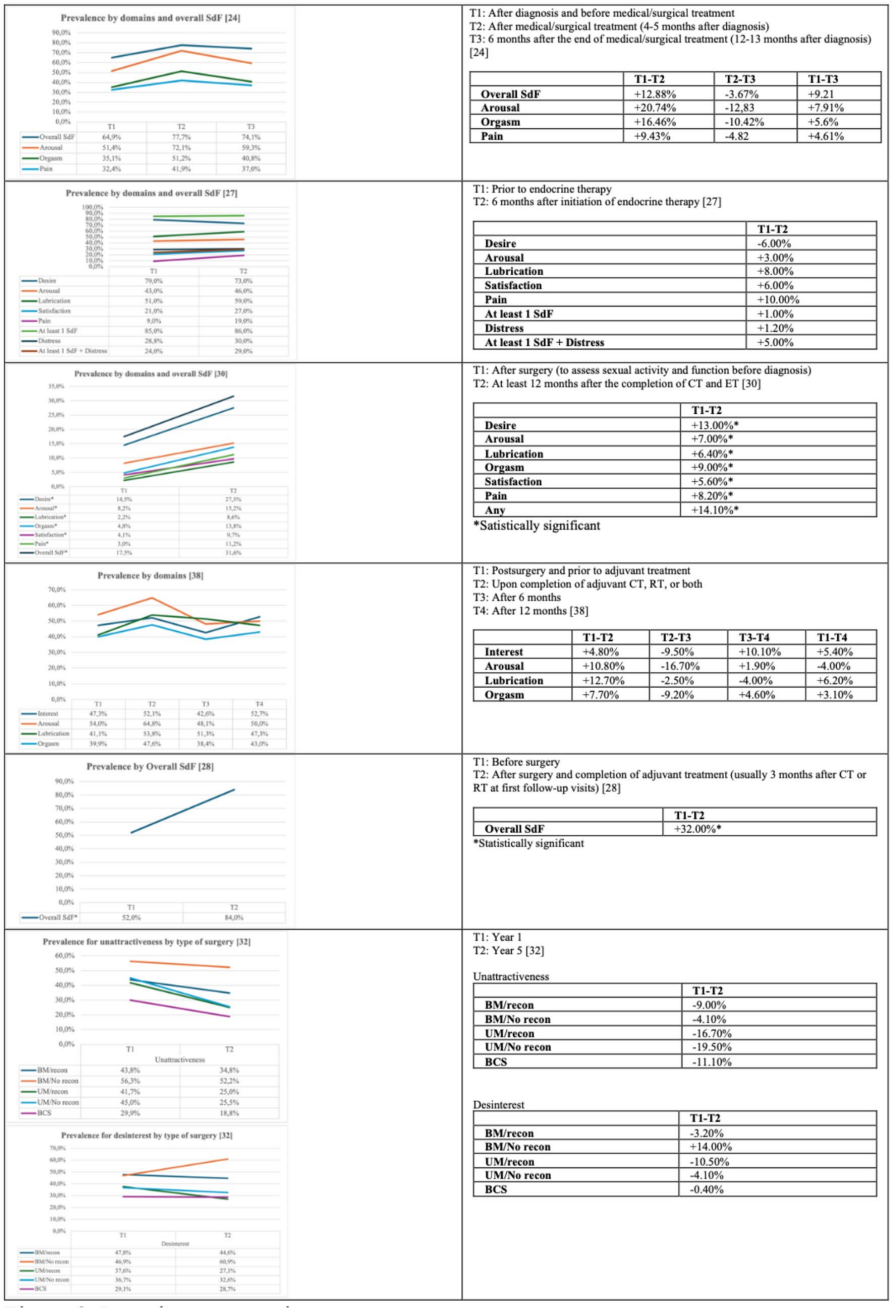
Changes in prevalence of sexual dysfunction over time

Prevalence rates for SdF were reported in four studies [24, 27, 28, 30]. Prevalence rates ranged from 17.5% at the pretreatment evaluation to 86.0% after 6 months of hormone therapy (HT), exceeding 50% at seven time points. Prevalence increased over time in all four studies, but was only statistically significant in two studies [28, 30] (Fig. 2). One study [27] reported both prevalence and incidence data, with an SdF incidence rate of 7.6% and a sexual distress incidence of 11% after 6 months of HT.

**Fig. 3:**
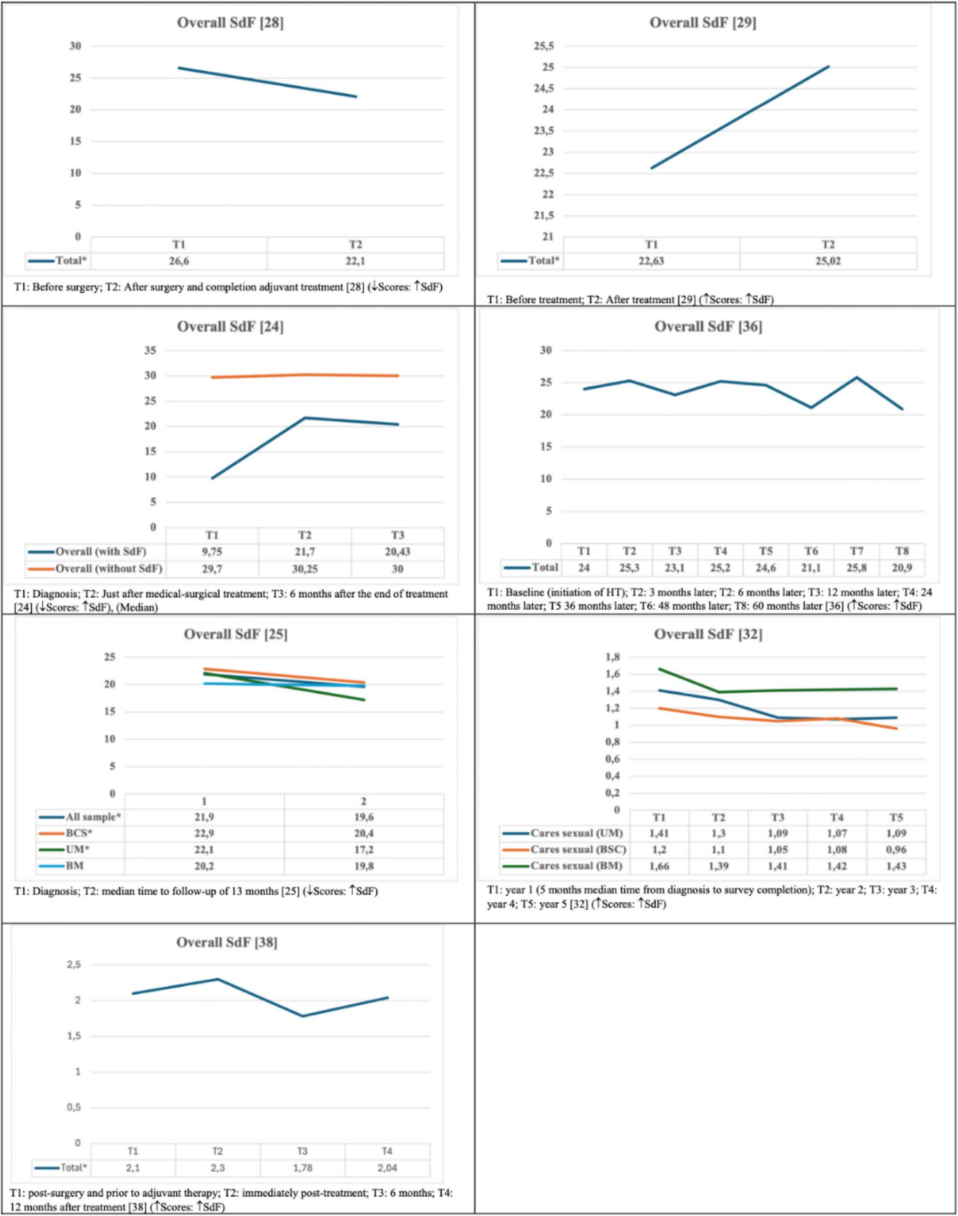
Overall sexual dysfunction scores

Seven studies reported mean or median SdF scores [24 25, 28, 29, 32, 36, 38]. Of those seven studies, four reported statistically significant changes across some time points. Three studies showed that SdF worsened following treatment, with increases of 10.5%, 10.6%, and 17.0% in mean scores, respectively [25, 28, 29]. One study found that SdF fluctuated over time [38]. Three studies found no statistically significant changes [24, 32, 36] (Fig. 3).

Numerous factors were associated with a greater risk of SdF, including pretreatment SdF [27, 28], HT [28], anxiety, partnered status, ovarian suppression/removal, tumour stage, severe musculoskeletal pain, body image issues, obesity [37], and worsening in endocrine symptoms [36]. None of the following variables was associated with SdF: educational level; employment status; ChT; radiotherapy; breast conserving surgery [28]; physical function; prior unilateral mastectomy [36]; or gynaecological symptoms [27]. No clear association was found between age and SdF, with one study finding an association between younger age and worse SdF outcomes [28] and two other studies finding no significant association [27, 30]. ChT emerged as a potential protective factor in one study [36] (Online Resource S3).

#### Desire

Two studies reported the prevalence of disturbances in sexual desire [24, 30], which ranged from 14.5 before surgery to 79% prior to HT. One of those studies showed a statistically significant increase in low desire (from 14.5 to 27.5%) [30], while the other study reported a non-significant change [24] (Fig. 2).

**Fig. 4:**
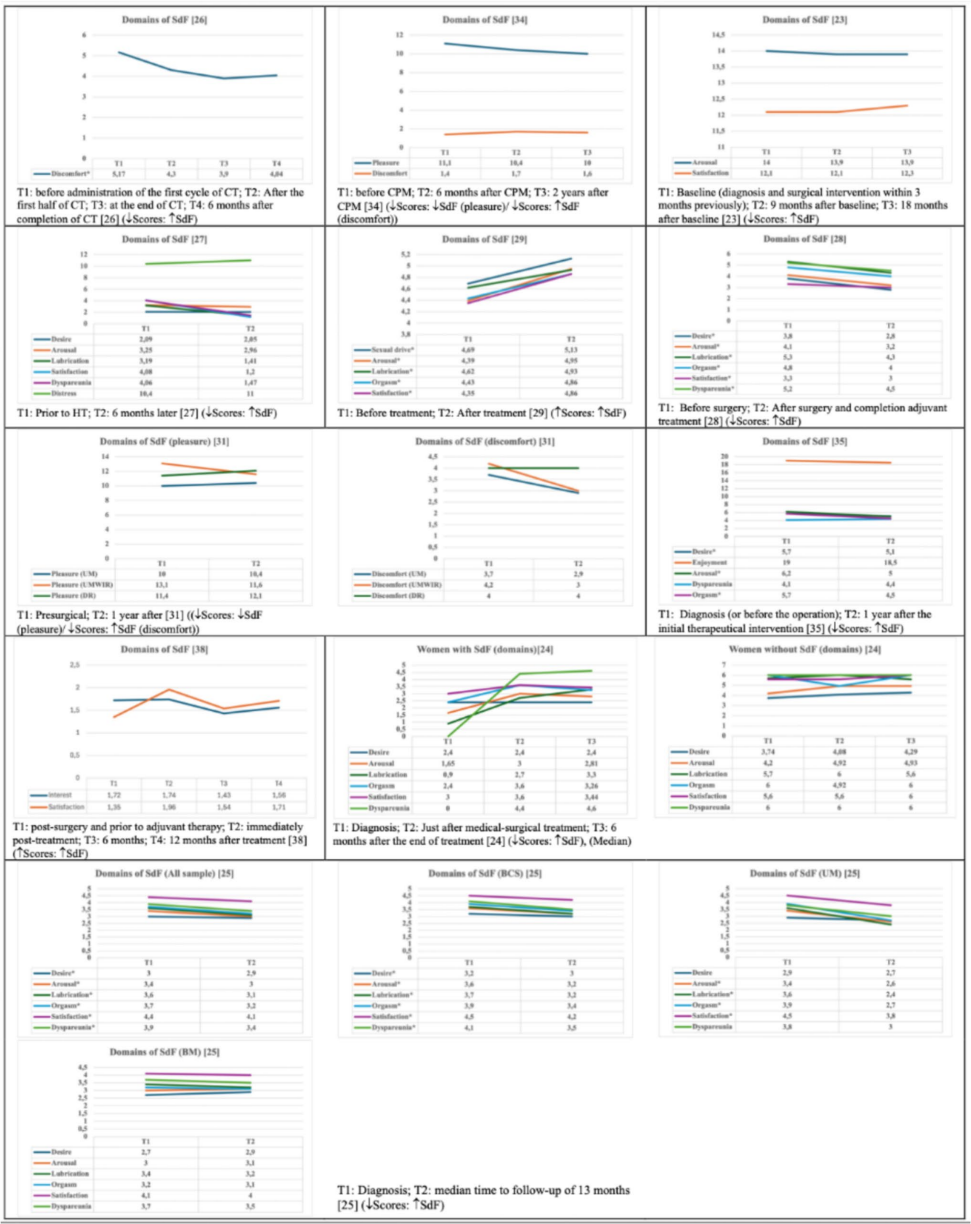
Sexual dysfunction domain scores

Five studies reported desire scores [24, 25, 27, 28, 35], with three reporting statistically significant increases in alterations in sexual desire following treatment, with increase of 3.3%, 10.5%, and 26.3% in mean scores, respectively [25, 28, 35]. The other two studies found no significant changes [24, 27] (Fig. 4).

ChT-induced menopause correlated with low desire (odds ratio [OR] = 2.8, *p* < 0.05). By contrast, none of the following variables was associated with desire: age; marital status; current HT; type of surgery; depression; time since diagnosis; previous gonadotropin-releasing hormone (GnRH) treatment; and comorbidities [30] (Online Resource S3).

#### Arousal

Four studied provided data on the prevalence of arousal disorder [24, 27, 30, 38], which ranged from 8.2% (pre-diagnosis) to 72.1% after medical/surgical treatment (4–5 months after diagnosis). One study found a statistically significant increase (7%) in this disorder [30], and two reported no significant changes [24, 38] (Fig. 2).

Seven studies provided arousal scores [23–25, 27–29, 35]. Of these, four found significant worsening in arousal scores following treatment (increases of 11.8%, 12.8%, 19.4%, and 22.0% in mean scores, respectively) [25, 28, 29, 35], while three found no significant changes [23, 24, 27] (Fig. 4).

The reported risk factors were menopausal at diagnosis, baseline depression [23], and ChT-induced menopause [30]. Age, education, and marital status yielded mixed results [23, 30]. Occupation, parity, income, and concomitant illness showed no association with arousal [23, 30] (Online Resource S3).

#### Lubrication

Prevalence rates for lubrication disorder were reported in three studies [27, 30, 38], which ranged from 2.2% before diagnosis to 59.0% after 6 months of HT. One study found a statistically significant increase of 6.4% [30], while two studies showed non-significant changes [24, 27] (Fig. 2).

Five studies provided lubrication scores [24, 25, 27–29], with three reporting a statistically significant worsening in lubrication following treatment, with increases of 6.7%, 13.9%, and 18.9%, respectively, in mean scores [25, 28, 29]. The other two studies found no significant association between treatment and lubrication status [24, 27] (Fig. 4).

ChT-induced menopause was associated with low lubrication (OR = 4.7, *p* < 0.05). None of the following variables was associated with lubrication status: age; marital status; education; occupation; parity; current HT; type of breast surgery; depression; time since diagnosis; previous GnRH treatment; and comorbidities [30] (Online Resource S3).

#### Dyspareunia

Three studies provided prevalence data for dyspareunia [24, 27, 30], which ranged from 3% before diagnosis to 41.9% after medical/surgical treatment. One study presented statistically significant differences with an 8.2% increase [30], while two studies found no significant chances [24, 27] (Fig. 2).

Five studies evaluated dyspareunia scores [24, 25, 27, 28, 35]. Of these, two studies found statistically significant worsening after treatment (11.3% and 12.9%: percentage increases in mean values) [25, 28] and three studies found no significant changes [24, 27, 35] (Fig. 4).

ChT-induced menopause was significantly associated with post-treatment dyspareunia. However, no significant associations were reported between dyspareunia and any of the following variables: age; marital status; education; occupation; parity; current HT; type of surgery; depression; time since diagnosis; previous GnRH treatment; and comorbidities [30] (Online resource S3).

#### Orgasm

The prevalence of orgasm disorder was evaluated in three studies [24, 30, 38], which reported prevalence rates ranging from 4.8% before diagnosis to 51.2% after medical/surgical treatment. Of those three studies, only one found a statistically significant increase (9%) [30], while the other did not find any significant changes [24, 38] (Fig. 2).

Five studies evaluated orgasm disorder scores [24, 25, 28, 29, 35]. Of those, four showed statistically significant worsening after treatment, with a reported 9.7%, 13.5%, 16.7%, and 21.1% increases in mean values, respectively [25, 28, 29, 35]. The remaining study found no significant changes [24] (Fig. 4).

ChT-induced menopause was associated with low orgasm (OR = 5.46, *p* < 0.05). None of the following variables was significantly associated with orgasm disorders: age; marital status; education; occupation; parity; current HT; type of breast surgery; depression; time since diagnosis; previous GnRH treatment; and presence of comorbidities [30] (Online resource S3).

#### Satisfaction

Two studies assessed the prevalence of satisfaction disorder [27, 30], with reported prevalence rates ranging from 4.1% before diagnosis to 27% after six months of HT. One of the studies reported a statistically significant increase (6.6%) in this disorder [30] while the other found no significant changes [27] (Fig. 2).

Six studies reported satisfaction disorder scores [23–25, 27–29], with three finding a statistically significant increase (worsening) after treatment (6.8%, 9.1%, and 11.7%, respectively) [25, 28, 29] and the other three reporting no significant changes [23, 26, 27] (Fig. 4).

These studies described several potential risk factors associated with low sexual satisfaction including the following: emotional closeness with partner [33]; baseline depression, no mastectomy [23]; days out of role due to disability; menopause symptoms; mood disorder [38]; and ChT-induced menopause [30]. No significant associations were observed between low sexual satisfaction and any of the following variables: age, education, type of surgery [23, 30]; marital status; occupation; parity; time since diagnosis; past GnRH treatment; current or past HT [30]; married/living as married, premenopausal at baseline, income [23]; and depression [30, 33]. The presence of comorbidities showed mixed results [23, 30, 33] (Online Resource S3).

#### Other SdF domains

The following domains were only assessed in a few studies: unattractiveness, discomfort, pleasure/enjoyment, distress, sexual drive, and sexual interest [26, 27, 29, 31, 32, 34, 35, 38]. One study reported statistically significant increase in sexual discomfort during and after completion of ChT. However, discomfort improved at 6 months post-ChT in the whole sample and in the subgroup of premenopausal women [26]. Another study found a significant decrease in sexual drive from pre-treatment levels to the post-treatment assessment [29]. No significant differences were observed in the other domains evaluated in these studies.

## Discussion

To the best of our knowledge, this systematic review is the first to comprehensively assess the incidence and prevalence of SdF in women diagnosed with BC, including a description of changes in SdF over time and potential risk factors. A total of 16 longitudinal cohort studies were included in this review. Those studies were highly heterogeneous in terms of study design, evaluation instruments (11 different tools were used to evaluate SdF), the timing of assessments, and the characteristics of the cohorts. Given the large variability in the design of those studies and in the reported results, we were unable to pool results. The most commonly evaluated SdF domains were desire, arousal, lubrication, orgasm, satisfaction, and dyspareunia, largely because these domains are included in the FSFI, the most widely used scale due to its reliability and ease of use [39]. In terms of methodological quality (Table 1), most of the studies were of moderate quality.

The scant data on SdF incidence rates is concerning, as it limits our ability to precisely characterize the onset and development of SdF in women undergoing treatment for BC, which is essential to establish the temporal sequence of this disorder and thus to determine a causal relationship. To our knowledge, there are no published studies on the incidence of SdF in women with BC, thus limiting our ability to compare our results to other studies. Moreover, several primary studies and reviews have confused prevalence with incidence. As McCabe et al. observed, there is a notable scarcity of studies on the incidence of SdF in women (healthy or otherwise). In addition, the available studies are highly heterogenous in terms of data collection strategies leading those authors to emphasize the need for a clearly defined data collection strategy in future research [40].

Our findings suggest that the prevalence of SdF tends to increase in the short term (i.e., approximately one year after diagnosis or treatment completion), thus underscoring the heightened vulnerability of women with BC to SdF. These findings are consistent with other systematic reviews, despite differences in inclusion criteria. For example, one review evaluated studies that used the FSFI to assess SdF in women with BC, reporting a pooled prevalence of 73% (95% CI, 64.0–82.8%) [16]. Another systematic review of seven primary studies—most of which were cross-sectional and comprised of BC survivors in Arab countries—also reported high prevalence ranges in several SdF domains, including dyspareunia, lubrication, desire, orgasm, and arousal [17].

We found that several SdF domains exhibited a consistent pattern of progressive deterioration over time. Unfortunately, we cannot directly compare our results to other studies due to the scarcity of comparable longitudinal research studies that have used validated instruments. A longitudinal study in patients with metastatic BC found that mean SdF severity scores increased over time, in line with our results [41]. However, that study did not use validated scales. The magnitude of the changes observed in that study (often > 10%) indicates the presence of clinically significant impairments that should be addressed as part of survivorship care. Those findings are in line with a systematic review that assessed the association between BC survivorship and adverse mental health outcomes, which found strong evidence of an increased risk of SdF across various domains in women with BC compared to those without a cancer diagnosis [42].

Although our review included only a limited number of well-designed cohort studies, our findings reveal a broad range of potential risk factors for SdF in BC survivors. This complex interplay of contributing elements underscores the intricate aetiology of SdF, a finding that is consistent with previous reports [17, 41]. The aetiology appears to be influenced by a constellation of factors, including the physical sequelae of BC treatments, psychological distress, comorbidities, and lifestyle factors. Sociocultural influences, such as gender taboos, subjective norms, and hidden values in sexual relationships, also appear to play a significant role, as these factors may directly and indirectly impact the sexual health in this patient population [43].

Several studies in this review found that age, marital status, and educational level were potential risk factors for SdF; however, these associations were not consistent across the studies. SdF may manifest in different ways depending on the age group due to differences in life stage, hormonal changes, and personal expectations. While younger women appear to experience sexual problems and reproductive concerns [44], older age has also been identified as a risk factor for SdF [9]. Younger women might find it more difficult to accept treatment-related sexual problems while older women may be more likely to accept age-related changes in sexual function, leading them to prioritize other aspects of well-being. It is worth noting, however, that some studies have not found any significant association between SdF and age [45].

The type and intensity of treatment is a key factor that can directly impact sexual function through physical changes (e.g., surgery) or indirectly via treatment-related side effects (e.g., ChT-induced menopause), which influence both QoL and sexual function. HT, ChT-induced menopause, and ovarian suppression were all identified as significant risk factors for SdF, with ChT-induced menopause affecting multiple SdF domains. This is especially concerning for younger women, who report concerns about the impact of treatment on sexual function [44] and fertility [46].

Several of the studies identified mental health issues (e.g., depression and anxiety) as potential risk factors for SdF. The post-diagnosis emotional and psychological challenges can significantly affect mental health, and, in turn, sexual function. Obesity and other body image-related factors, which are often interconnected, were also relevant risk factors for SdF. Negative body image may contribute to emotional distress and may even lead to depression, which in turn can worsen sexual function. Surgical treatments can also negatively impact patients’ perceived femininity and attractiveness, a finding that underscores the importance of addressing both psychological well-being and body image to improve sexual health outcomes in this patient population. Previous studies have shown that both depression [47] and body image [9] can negatively influence SdF in women with BC, which is why it is crucial to address these factors in the care process.

Several other health-related factors (concomitant illnesses, days of disability, and severe musculoskeletal pain) were potential risk factors for SdF, a finding that suggests that broader health challenges can exacerbate SdF. These factors likely contribute to physical discomfort, reduced mobility, and chronic pain, all of which can affect sexual function. Additionally, extended periods of disability may lead to decreased self-esteem and psychological distress, further undermining sexual function. The negative impact of these health burdens underscores the importance of addressing the overall health and QoL of women with BC in order to improve sexual well-being.

There is a notable lack of high-quality, prospective cohort-controlled studies in women without SdF, despite the increased likelihood of developing SdF after menopause. Consequently, there is a clear need for more research to better understand and characterise the early development of SdF. At present, most studies rely solely on prevalence data, and thus the specific contributing factors to SdF may be overlooked. Future studies should stratify participants by subgroups (e.g., by treatment type) to identify distinct sexual health patterns, which may allow for more tailored interventions. Additionally, more research is needed to elucidate the complex interactions between and among biopsychosocial factors to fully understand their cumulative impact on SdF.

## Strengths and limitations

This systematic review has several limitations. First, we were unable to perform a meta-analysis of the data due to the substantial heterogeneity in study designs, assessment tools, and follow-up times. In this regard, it would be desirable if future research studies utilized standardised methodologies and consistent outcome measures to enable more robust comparisons between studies and to allow for meta-analytic approaches. Second, only one study included in this review reported incidence rates, which necessarily limits our ability to understand the onset of SdF in BC survivors. Third, given the moderate methodological quality of these studies, the reported results may have been biased by confounders, which may reduce the reliability of our review. Finally, there may also be a risk of publication bias, as studies with non-significant findings may be underrepresented in the literature.

In this review, we focused on longitudinal studies to assess changes in mean SdF scores over time. However, due to the numerous methodological limitations of these studies, we were unable to estimate the incidence of SdF and certain some risk factors with a high degree of certainty. We identified a wide range of physical, psychological, and sociodemographic risk factors that contribute to SdF, a finding that reinforces the multifactorial nature of SdF while also providing a strong foundation for future research. Moreover, our findings underscore the importance of integrating the assessment of SdF into survivorship care protocols. An important strength of this study is the inclusion of data from studies conducted in diverse geographical regions, thus providing a more comprehensive understanding of SdF in women with BC in different regions and contexts. However, due to the lack of data, we were not able to assess how the cultural, social, and economic differences in the patients included in those studies may have influenced the perception and characteristics of SdF.

## Conclusions

The findings of this systematic review highlight the high prevalence of SdF, particularly in the first year after diagnosis, in women with BC. This finding supports the routine assessment of SdF as part of survivorship care. Due to the lack of data on incidence rates (only one study provided these data), our understanding of the onset of SdF is limited. Moreover, it is difficult to compare the onset of SdF in women with and without BC given the scant data currently available. This review shows that many different risk factors may be associated with SdF. However, more research is needed to determine the variables that have the greatest impact on SdF in this patient population. More specifically, there is a clear need for high-quality, longitudinal cohort-controlled studies with standardised assessment tools to determine the incidence and changes in SdF over time.

## Supplementary Information

Below is the link to the electronic supplementary material.

Supplementary file1 (PDF 378 KB)

Supplementary file2 (PDF 110 KB)

Supplementary file3 (PDF 119 KB)

## Data Availability

No datasets were generated or analysed during the current study.
