# Supplementary material for: Sexual dysfunction in women with breast cancer: a systematic review
Source: Support Care Cancer. 2025 Mar 31;33(4):332. doi: 10.1007/s00520-025-09352-6 (PMC11958476; doi:10.1007/s00520-025-09352-6)
Supplement: Supplementary file 1 — Supplementary file1 (PDF 378 KB) [file 520_2025_9352_MOESM1_ESM.pdf]

S1. MEDLINE (via PubMed) Search

PubMedSearchHistory-3

Search number	Query	Sort By	Filters
31	#5 AND #29		from 2010/1/1 - 2023/11/30
30	#5 AND #29		
29	#6 OR #7 OR #8 OR #9 OR #10 OR #11 OR #12 OR #13 OR #14 OR #15 OR #16 OR #17 OR #18 OR #19 OR #20 OR #21 OR #22 OR #23 OR #24 OR #25 OR #26 OR #27 OR #28		
28	"sexual life"[Title/Abstract]		
27	"sexual well*"[Title/Abstract]		
26	sexual*[Title]		
25	psychosexual[Title/Abstract]		
24	sexuality[Title/Abstract]		
23	"orgasmic disorders"[Title/Abstract:~3]		
22	"orgasmic dysfunction"[Title/Abstract:~3]		
21	"orgasmic disorder"[Title/Abstract:~3]		
20	dyspareunia[Title/Abstract]		
19	"painful intercourse"[Title/Abstract:~3]		
18	"sex painful"[Title/Abstract:~3]		
17	"sexual painful"[Title/Abstract:~3]		
16	"sex pain"[Title/Abstract:~3]		
15	"sexual pain"[Title/Abstract:~3]		
14	sexual arousal[Title/Abstract]		
13	sexual desire[Title/Abstract]		
12	sexual interest*[Title/Abstract]		
11	sexual function*[Title/Abstract]		
10	sexual dysfunction*[Title/Abstract]		
9	sexual activit*[Title/Abstract]		
8	"Sexual Dysfunctions, Psychological"[MeSH Terms]		
7	"Sexual Dysfunction, Physiological"[MeSH Terms]		
6	"Sexuality"[MeSH Major Topic]		
5	#1 OR #2 OR #3 OR #4		
4	mastectom*[Title/Abstract]		
3	"breast reconstruct*"[Title/Abstract]		
2	breast cancer[Title/Abstract]		
1	"Breast Neoplasms"[MeSH Terms]		

Search Details	Results	Time
(("Breast Neoplasms"[MeSH Terms] OR "breast cancer"[Title/Abstract] OR "breast reconstruct*"[Title/Abstract] OR "mastectom*"[Title/Abstract]) AND ("Sexuality"[MeSH Major Topic] OR "sexual dysfunction, physiological"[M	1,595	03:15:23
("Breast Neoplasms"[MeSH Terms] OR "breast cancer"[Title/Abstract] OR "breast reconstruct*"[Title/Abstract] OR "mastectom*"[Title/Abstract]) AND ("Sexuality"[MeSH Major Topic] OR "sexual dysfunction, physiological"[Me	2,222	03:12:15
"Sexuality"[MeSH Major Topic] OR "sexual dysfunction, physiological"[MeSH Terms] OR "sexual dysfunctions, psychological"[MeSH Terms] OR "sexual activit*"[Title/Abstract] OR "sexual dysfunction*"[Title/Abstract] OR "se:	184,448	03:11:49
"sexual life"[Title/Abstract]	2,867	03:09:23
"sexual well*"[Title/Abstract]	968	03:09:04
"sexual*"[Title]	104,332	03:08:46
"psychosexual"[Title/Abstract]	2,234	03:08:31
"sexuality"[Title/Abstract]	19,290	03:08:18
"orgasmic disorders"[Title/Abstract:~3]	81	03:08:04
"orgasmic dysfunction"[Title/Abstract:~3]	255	03:07:46
"orgasmic disorder"[Title/Abstract:~3]	140	03:07:19
"dyspareunia"[Title/Abstract]	5,009	03:06:53
"painful intercourse"[Title/Abstract:~3]	245	03:06:39
"sex painful"[Title/Abstract:~3]	83	03:06:16
"sexual painful"[Title/Abstract:~3]	147	03:05:59
"sex pain"[Title/Abstract:~3]	2,311	03:05:37
"sexual pain"[Title/Abstract:~3]	1,988	03:05:23
"sexual arousal"[Title/Abstract]	2,497	02:59:59
"sexual desire"[Title/Abstract]	3,919	02:59:22
"sexual interest*"[Title/Abstract]	1,483	02:59:06
"sexual function*"[Title/Abstract]	17,798	02:58:50
"sexual dysfunction*"[Title/Abstract]	14,112	02:58:19
"sexual activit*"[Title/Abstract]	14,717	02:57:56
"sexual dysfunctions, psychological"[MeSH Terms]	30,398	02:57:06
"sexual dysfunction, physiological"[MeSH Terms]	33,729	02:53:51
"Sexuality"[MeSH Major Topic]	31,012	02:50:23
"Breast Neoplasms"[MeSH Terms] OR "breast cancer"[Title/Abstract] OR "breast reconstruct*"[Title/Abstract] OR "mastectom*"[Title/Abstract]	459,220	02:49:36
"mastectom*"[Title/Abstract]	28,482	02:49:01
"breast reconstruct*"[Title/Abstract]	12,357	02:48:44
"breast cancer"[Title/Abstract]	345,275	02:48:06
"Breast Neoplasms"[MeSH Terms]	346,941	02:47:39
