# Supplementary material for: Sexual dysfunction in women with breast cancer: a systematic review
Source: Support Care Cancer. 2025 Mar 31;33(4):332. doi: 10.1007/s00520-025-09352-6 (PMC11958476; doi:10.1007/s00520-025-09352-6)
Supplement: Supplementary file 2 — Supplementary file2 (PDF 110 KB) [file 520_2025_9352_MOESM2_ESM.pdf]

S2. Quality assessment of the included studies

	Q1	Q2	Q3	Q4	Q5	Q6	Q7	Q8	Q9	Q10	Q11	Total
Harirchi et al. [28]	NA	NA	Y	U	Y	N	Y	Y	Y	Y	Y	63.6%
Bober et al. [23]	NA	NA	Y	U	Y	N	Y	Y	Y	Y	Y	63.6%
Farthmann et al. [26]	Y	Y	N	U	Y	N	Y	Y	Y	Y	Y	72.2%
Lee et al. [30]	NA	NA	Y	U	Y	U	Y	Y	Y	Y	Y	63.6%
Unukovych et al. [34]	NA	NA	Y	U	N	U	Y	Y	Y	Y	Y	54.5%
İzci et al. [29]	Y	Y	Y	U	Y	U	Y	Y	Y	Y	Y	81.8%
Frechette et al. [27]	NA	NA	Y	U	Y	N	Y	Y	Y	Y	Y	63.6%
Vaidakis et al. [35]	Y	Y	Y	U	Y	U	Y	Y	Y	Y	Y	81.8%
Córdoba-de Juan et al. [24]	NA	NA	Y	U	N	N	Y	Y	Y	N	Y	45.5%
Cornell et al. [25]	Y	Y	Y	U	N	N	Y	Y	Y	Y	Y	72.2%
vonHippel et al. [37]	NA	NA	Y	U	Y	N	Y	Y	Y	N	Y	54.5%
Metcalfe et al. [31]	Y	Y	Y	U	Y	U	Y	Y	Y	N	Y	72.2%
Webber et al. [38]	NA	NA	Y	U	Y	N	Y	Y	Y	N	Y	54.5%
Rosenberg et al. [32]	Y	Y	Y	U	Y	N	Y	Y	Y	U	Y	72.2%
Rottmann et al. [33]	NA	NA	Y	U	Y	U	Y	Y	Y	Y	Y	63.6%
Verma et al. [36]	NA	NA	Y	U	Y	N	Y	Y	Y	N	Y	54.5%

NA: Not Applicable; Y: Yes; U: Unclear; N: Not

Moola S, Munn Z, Tufanaru C, Aromataris E, Sears K, Sfetcu R, Currie M, Qureshi R, Mattis P, Lisy K, Mu P-F. Chapter 7: Systematic reviews of etiology and risk. In: Aromataris E, Munn Z (Editors). JBI Manual for Evidence Synthesis. JBI, 2020. Available from https://synthesismanual.jbi.global

Q1. Were the two groups similar and recruited from the same population?

Q2. Were the exposures measured similarly to assign people to both exposed and unexposed groups?

Q3. Was the exposure measured in a valid and reliable way?

Q4. Were confounding factors identified?

Q5. Were strategies to deal with confounding factors stated?

Q6. Were the groups/participants free of the outcome at the start of the study (or at the moment of exposure)?

Q7. Were the outcomes measured in a valid and reliable way?

Q8. Was the follow up time reported and sufficient to be long enough for outcomes to occur?

Q9. Was follow up complete, and if not, were the reasons to loss to follow up described and explored?

Q10. Were strategies to address incomplete follow up utilized?

Q11. Was appropriate statistical analysis used?
