# Supplementary material for: Sexual dysfunction in women with breast cancer: a systematic review
Source: Support Care Cancer. 2025 Mar 31;33(4):332. doi: 10.1007/s00520-025-09352-6 (PMC11958476; doi:10.1007/s00520-025-09352-6)
Supplement: Supplementary file 3 — Supplementary file3 (PDF 119 KB) [file 520_2025_9352_MOESM3_ESM.pdf]

S3. Potential risk factors

S 3.1. Sociodemographic factors

Study	Time points assessed	Age	Marital status/ partnership	Others
Harirchi et al., 2012 [28]	Before surgery, after surgery and completion of adjuvant treatment	Younger age more SdF at post treatment (OR=0.95, 95%CI [0.93-0.98], p=0.04		- Education NS for SdF (MR)<br>- Employment NS for SdF (MR)
Lee et al., 2015 [30]	After surgery (to assess/ask before diagnosis) and at least 12 months after the completion of CT or HT (after treatment)<br>Median: 30.0 months from diagnosis	NS for desire, arousal, lubrication, orgasm, satisfaction, pain (MR)	Marital status NS for desire, arousal, lubrication, orgasm, satisfaction, pain (MR)	- Education NS for all domains (MR)<br>- Occupation NS for all domains (MR)<br>- Parity NS for all domains (MR)
Bober et al., 2013 [23]	Baseline (DCIS diagnosis and surgical intervention within 3 months previously), 9 and 18 months after baseline	- Younger (<50 years) better arousal scores than older (>65 years). (RIM random intercept model)<br>- Satisfaction: NS (RIM)	Married/living as married less arousal scores (more problems) compared to those who were not (RIM)<br>Satisfaction NS (RIM)	- high school diploma or less: better arousal scores (RIM)<br>- Education: NS for satisfaction (RIM)<br>- Income NS for arousal or satisfaction (RIM)
Frechette et al., 2013 [27]	Prior to HT (T1), and 6 months later (T2)	NS for SdF (MR)		
Von Hippel et al., 2019 [37]	Diagnosis, 1-year from diagnosis, 2 years, 3-year, 4-year, and 5-year from diagnosis		Stable mild (RRR: 3.05, 95% CI [1.40-6.65], p=0.01/ worsening moderate (RRR=9.22, 95%CI [2.21-38.40], p=0.00/ improving moderate (RRR=6.83, 95%CI [2.60-17.89], p=0.00/ Stable severe (RRR=10.35, 95%CI [2.77-38.63], p=0.00, all more likely to be partnered than women in the stable asymptomatic trajectory.	
Webber et al., 2011 [38]	Post-surgery and prior to adjuvant therapy, immediately post-treatment, 6 months and 12 months after treatment		Marital status was associated with sexual interest at end of treatment (B=1.08) p<0.05	
Rottmann et al., 2017 [33]	T1 (baseline, </= 4 months following BC surgery), T2 (five months later)		More emotional closeness of the partner, more sexual satisfaction (B=1.97, 95%CI [0.71-3.22], p<0.01 (multilevel model)	

S 3.2. Treatment factors

Study	Time points assessed	HT	CT	Surgery	Radiotherapy	Others
Bober et al., 2013 [23]	Baseline (DCIS diagnosis and surgical intervention within 3 months previously), 9 and 18 months after baseline	- NS for arousal (RIM)<br>- NS for satisfaction (RIM)		- Arousal: NS (RIM)<br>- Satisfaction: better satisfaction scores for mastectomy (with or without reconstruction) than no mastectomy (RIM)		
Harirchi, et al., 2012 [28]	Before surgery, after surgery and completion of adjuvant treatment	Receiving HT more SdF at post-treatment (OR (MR)=3.34, 95% CI [1.38-8.06], p=0.007)	CT NS for SdF (MR)	- Conservative *vs* Mastectomy NS for SdF (MR)	RT NS for SdF (MR)	- Poor sexual dysfunction at pre-treatment more SdF at post-treatment (OR (MR)=12.3, 95% CI [3.39-39.0], p<0.0001)
Lee et al., 2015 [30]	After surgery (to assess/ask before diagnosis) and at least 12 months after the completion of CT or HT (after treatment)<br><br>Median: 30.0 months from diagnosis	Current HT NS for desire, arousal, lubrication, orgasm, satisfaction and pain (MR)	- CT-related menopause more likely to experience low desire (OR=2.8, 95% CI [1.26-6.27], p<0.05),<br>low arousal (OR=4.81, 95% CI [1.53-15.18], p<0.05), low lubrication after treatment (OR=4.68, 95% CI [1.27-17.23], p<0.05) low orgasm (OR=5.46, 95% CI [1.75-16.99], p<0.05), low satisfaction (OR=4.53, 95% CI [1.24-16.55], p<0.05) and sexual pain (OR=3.52, 95% CI [1.05-11.82], p<0.05) after treatment	- Type of breast surgery NS for desire, arousal, lubrication, orgasm, satisfaction and pain (MR)		
Frechette et al., 2013 [27]	Prior to HT (T1), and 6 months later (T2)	Women experiencing SdF at T1 more likely to experience SdF at T2 (OR=7.410, 95% CI [1.487-36.917], p=0.015)				
Von Hippel et al., 2019 [37]	Diagnosis, 1-year from diagnosis, 2 years, 3-year, 4-year, and 5-year from diagnosis		Women in the worsening moderate trajectory significantly less likely to have received chemotherapy (RRR=0.33, 95%CI [0.13-0.83], p=0.02) than women in the stable asymptomatic trajectory			
Verma et al., 2022 [36]	Baseline (initiation of HT), 3, 6, 12, 24, 36, 48, 60 months later			Prior mastectomy NS for SdF (MR)		

S 3.3. Menopause factors

Study	Time points assessed	Premenopausal	Postmenopausal	Others
Bober et al., 2013 [23]	Baseline (DCIS diagnosis and surgical intervention within 3 months previously), 9 and 18 months after baseline	- Menopausal at baseline lower arousal scores (RIM)<br>- Satisfaction: NS (RIM)		

S 3.4. Mental health factors

Study	Time points assessed	Anxiety	Depression	Others
Bober et al., 2013 [23]	Baseline (DCIS diagnosis and surgical intervention within 3 months previously), 9 and 18 months after baseline		- Not depressed at baseline better arousal scores (RIM)<br>Not depressed at baseline better satisfaction scores (RIM)	
Lee et al., 2015 [30]	After surgery (to assess/ask before diagnosis) and at least 12 months after the completion of CT or HT (after treatment)<br>Median: 30.0 months from diagnosis		NS for desire, arousal, lubrication, orgasm, satisfaction, and pain (MR)	
Von Hippel et al., 2019 [37]	Diagnosis, 1-year from diagnosis, 2 years, 3-year, 4-year, and 5-year from diagnosis	Stable severe more likely to experience anxiety than stable asymptomatic trajectory (RRR=3.11, 95%CI [1.16-8.31], p=0.02		
Webber et al., 2011 [38]	Post-surgery and prior to adjuvant therapy, immediately post-treatment, 6 month and 12 months after treatment			Mood disorder was independently associated with the overall sexual satisfaction item at pre-adjuvant treatment (B=1.04), end of treatment (B=1.36), 6 months (B=2.04) and 12 months (B=1.37) p<0.05.
Rottman et al., 2017 [33]	T1 (baseline, </= 4 months following BC surgery), T2 (five months later)		NS for satisfaction (multilevel model)	

S 3.4. Other factors

Study	Time points assessed	Other factors
Bober et al., 2013 [23]	Baseline (DCIS diagnosis and surgical intervention within 3 months previously), 9 and 18 months after baseline	- Concomitant illness that interferes with daily activities: negatively related to sexual satisfaction (RIM)<br>- Concomitant illness that interferes with daily activities: NS for arousal (RIM)
Lee et al., 2015 [30]	After surgery (to assess/ask before diagnosis) and at least 12 months after the completion of CT or HT (after treatment)<br>Median: 30.0 months from diagnosis	- Time since diagnosis NS for desire, arousal, lubrication, orgasm, satisfaction and pain (MR)<br>- Past GnRH NS for desire, arousal, lubrication, orgasm, satisfaction and pain (MR)<br>- Comorbidities NS for desire, arousal, lubrication, orgasm, satisfaction and pain (MR)
Frechette et al., 2013 [27]	Prior to HT (T1), and 6 months later (T2)	- Gynecological symptoms NS for SdF (MR)
Von Hippel et al., 2019 [37]	Diagnosis, 1-year from diagnosis, 2 years, 3-year, 4-year, and 5-year from diagnosis	- Stable mild (RRR: 2.70, 95% CI [1.43-5.12], p=0.00/ worsening moderate (RRR=11.85, 95%CI [5.34-26.29], p=0.00/ improving moderate (RRR=3.45, 95%CI [1.74-6.83], p=0.00/ Stable severe (RRR=12.40, 95%CI [5.39-28.52], p=0.00, all more likely to have undergone ovarian suppression/removal than women in the stable asymptomatic trajectory.<br><br>- Stage 2 was a significant predictor of membership in the worsening moderate (RRR=3.46, 95%CI [1.51-7.93], p=0.00) and stable severe (RRR=2.90, 95%CI [1.21-6.97], p=0.02) trajectories compared to stable asymptomatic<br><br>- Stable mild (RRR: 1.41, 95% CI [1.02-1.94], p=0.04/ improving moderate (RRR=2.06, 95%CI [1.46-2.89], p=0.00/ Stable severe (RRR=2.16, 95%CI [1.46-3.21], p=0.00, all more likely to experience severe musculoskeletal pain than women in the stable asymptomatic trajectory.<br><br>- Stable mild (RRR: 1.55, 95% CI [1.09-2.19], p=0.01/ worsening moderate (RRR=2.05, 95%CI [1.35-3.10], p=0.00/ improving moderate (RRR=2.01, 95%CI [1.39-2.90], p=0.00/ Stable severe (RRR=2.52, 95%CI [1.67-3.80], p=0.00, all more likely to report body image issues than women in the stable asymptomatic trajectory.<br><br>- Stable severe trajectory more likely to be obese than women in the stable asymptomatic trajectory (RRR=3.33, 95%CI [1.14-9.72], p=0.03
Webber et al., 2011 [38]	Post-surgery and prior to adjuvant therapy, immediately post-treatment, 6 month and 12 months after treatment	Days out of role due to disability was associated with sexual function at pre-adjuvant treatment (B=0.03), and 6 months (B=0.05), sexual interest at 6 months (B=0.03), and overall satisfaction at 6 months (B=0.05) p<0.05<br>Menopause symptoms were associated with overall satisfaction at 6 months (B=0.05) p<0.05
Rottman et al., 2017[33]	Post-surgery and prior to adjuvant therapy, immediately post-treatment, 6 month and 12 months after treatment	- Comorbidities NS for satisfaction (multilevel model)
Verma et al., 2022 [36]	Baseline (initiation of HT), 3, 6, 12, 24, 36, 48, 60 months later	-Physical function NS for SdF (MR)<br>Worsening in endocrine symptoms related to worsening in sexual problems OR=1.34, 95%CI [1.22-1.49], p<0.001

BC: Breast Cancer; BCS: Breast Conserving Surgery; BM: Bilateral Mastectomy; CI: Confidence Interval; CPM: Contralateral Prophylactic Mastectomy; CT: Chemotherapy; DCIS: Ductal Carcinoma In Situ; GnRH: Gonadotropin-releasing Hormone; HT: Hormonotherapy; MR: Multiple Logistic Regression; OR: Odds Ratio; NS: Not significant; RRR: Relative Risk Ratio: RT: Radiotherapy; SdF: Sexual Dysfunction; UM: Unilateral Mastectomy
